# Piperine as an Herbal Alternative for the Prevention of Drug-Induced Liver Damage Caused by Paracetamol

**DOI:** 10.3390/ph17111477

**Published:** 2024-11-02

**Authors:** Aline Meireles Coelho, Isabela Ferreira Queiroz, Luiza Oliveira Perucci, Tatiana Prata Menezes, Wanderson Geraldo Lima, André Talvani, Daniela Caldeira Costa

**Affiliations:** 1 Department of Biological Sciences (DECBI), Federal University of Ouro Preto (UFOP), Minas Gerais 35400-000, Brazil; alinemeireles0@yahoo.com.br (A.M.C.);; 2 Graduate Program in Biological Sciences (CBIOL), Federal University of Ouro Preto (UFOP), Minas Gerais 35400-000, Brazil; 3 Center for Research in Biological Sciences (NUPEB), Federal University of Ouro Preto (UFOP), Minas Gerais 35400-000, Brazil; 4 Graduate Program in Health and Nutrition (PPGSN), Federal University of Ouro Preto (UFOP), Minas Gerais 35400-000, Brazil

**Keywords:** acetaminophen, hepatic drug intoxication, hepatic protection, liver injury, paracetamol, piperine

## Abstract

**Background/Objective:** Hepatic drug intoxication is becoming increasingly common with the increasing use of chronic medications. Piperine has emerged as a promising alternative for protecting the liver against drug-induced injury. We evaluated the prophylactic effects of piperine in C57BL/6 mice with an acute liver injury induced by a paracetamol (APAP) overdose. **Methods:** Piperine was administered at a dose of 20 mg/kg (P20) or 40 mg/kg (P40) for eight consecutive days before the animals were exposed to a hepatotoxic dose of paracetamol (500 mg/kg). The animals were euthanized 3 h after the paracetamol overdose. **Results:** The prophylactic treatment with piperine (P20 and P40) maintained the levels of alanine aminotransferase (ALT) and the biomarkers of oxidative damage (TBARS and carbonylated proteins), which were statistically similar to those for the control group. The extent of hepatocyte necrosis and TNF-α (tumor necrosis factor-alpha) levels were lower than those in the group exposed to liver injury (APAP group). Piperine modulated the gene expression of CYP2E1 (cytochrome P4502E1) and the inflammasome pathway (NLRP3, CASP-1, IL-1β, and IL-18), which play a crucial role in the inflammatory response. In the P40 group, the degree of hepatic hyperemia was similar to that in the control group, as was the increase in metalloproteinase 9 (MMP-9) activity. **Conclusion:** Piperine has demonstrated beneficial and promising effects for the prevention of liver injury resulting from paracetamol-induced drug intoxication.

## 1. Introduction

The liver is the principal organ involved in xenobiotic metabolism. Hepatic drug intoxication is a common side effect of several medical treatments, including chemotherapy [1] and psychotropic [2,3,4,5], antifungal [6], anti-inflammatory [7], analgesic, and antipyretic drugs. These substances have the potential to elicit alterations in biochemical and inflammatory parameters, leading to a redox imbalance in the liver.

Among these drugs, paracetamol (N-acetyl-p-aminophenol; APAP, acetaminophen) has become the most widely used analgesic antipyretic in the United States [8], with an increased incidence of overdose in recent years [9,10,11], accounting for up to 48% of acute liver failure cases [12]. Thus, paracetamol remains a major cause of overdose, overdose-related liver failure, and death in the United States and many other countries [13,14].

Paracetamol is well known for causing acute liver intoxication, leading to alterations in the liver enzymes alanine aminotransferase (ALT) and aspartate aminotransferase (AST), oxidative stress, increased hepatocyte necrosis, and the dysregulation of inflammatory factors [15,16,17]. After an APAP overdose, its hepatotoxic metabolite N-acetyl-p-benzoquinone imine (NAPQI) depletes the antioxidant glutathione and binds to liver proteins, leading to mitochondrial dysfunction and increased reactive oxygen species (ROS) in the mitochondria [18]. The increase in ROS combined with the loss of glutathione results in a redox imbalance and oxidative stress [19].

The piperine content ranges from 1 to 2% in long pepper and from 5 to 10% in black pepper [20]. It has demonstrated a significant prophylactic potential owing to its hepatoprotective properties [21,22].

Piperine is a yellow crystalline substance with a molecular weight of 285.33 g/mol. Structurally, piperine can be divided into three components: the piperidine ring, aliphatic chain, and aromatic ring [23].

Scientific studies have described its various functions, including its antioxidant, anti-inflammatory, antipyretic, and gastroprotective properties, which can contribute to the prevention of various diseases [24,25,26]. Natural compounds, such as piperine, have proven to be valuable agents in the treatment of various pathologies. 

Previous studies conducted by our laboratory have evaluated piperine for the treatment of APAP overdose, using N-acetylcysteine (NAC) as the positive control. In one of the studies, the animals received an APAP overdose and, two hours later, were treated with a single dose of NAC and/or piperine. The goal of the study was to test the efficacy of piperine during the late phase of APAP-induced liver damage compared to that of the reference antidote NAC. Our results showed that the therapeutic effects of NAC could be potentiated by piperine in the treatment of paracetamol-induced hepatotoxicity [17]. 

In this study, we aimed to evaluate the potential of piperine to protect the liver from APAP-induced damage and assess its prophylactic effect when administered in successive doses during APAP metabolism. Based on this evidence, we hypothesized that enhancing the defense mechanisms of the body during drug intake could be an effective strategy with which to minimize or prevent liver damage. This study assessed the potential of piperine as a preventive herbal treatment against paracetamol-induced liver damage.

## 2. Results

### 2.1. Piperine Prophylaxis Reduces Liver Injury

**Figure 1:**
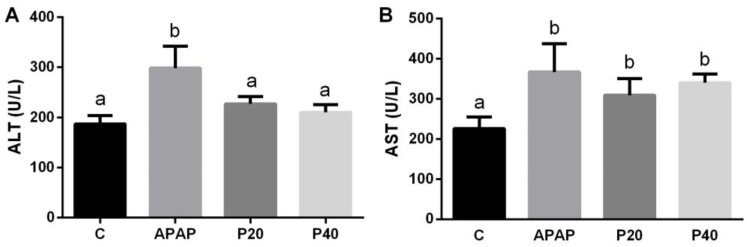
An evaluation of liver injury via ALT (**A**) and AST (**B**) levels in the serum of C57BL/6 mice subjected to prophylactic treatment with piperine (20 mg/kg and 40 mg/kg) for 8 days, followed by APAP intoxication. The different letters indicate statistical differences between the groups, where any letter other than “a” signifies a difference from the control group, and any letter other than “b” signifies a difference from the APAP group, as determined by a one-way analysis of variance followed by Tukey’s post hoc test. Statistical significance is considered at *p* < 0.05. ALT: alanine aminotransferase; AST: aspartate aminotransferase.

Figure 1A,B show a liver injury through the serum levels of the liver transaminases ALT and AST. We observed an increase in ALT activity in the APAP group compared with that in the control group. The piperine P20 (20 mg/kg) and P40 (40 mg/kg) groups were statistically similar to the control group (Figure 1A). A significant increase in the AST levels was observed in the APAP, P20, and P40 groups compared to that in the control group (Figure 1B), indicating that piperine did not prevent AST elevation. 

### 2.2. Piperine Prophylaxis Reduces Hepatic Necrosis

**Figure 2:**
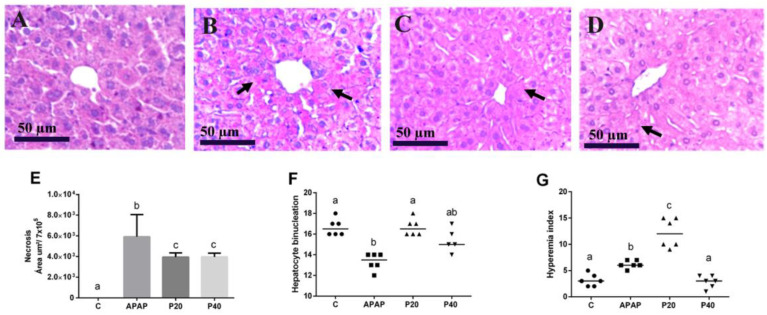
Histopathological evaluation and representative photomicrographs of the liver in C57BL/6 mice subjected to prophylactic piperine treatment for 8 days, followed by APAP intoxication. (**A**) The group treated with saline solution, showing normal liver architecture (control group); (**B**) the APAP group; and (**C**,**D**) the groups receiving piperine pretreatment of 20 mg/kg and 40 mg/kg, respectively. Staining: hematoxylin and eosin (HE). Magnification: 40x objective. The arrows indicate areas of necrosis. Panel (**E**) displays the percentage area of liver necrosis in the experimental groups, (**F**) shows the hepatocyte binucleation index, and (**G**) shows the hyperemia index. The different letters indicate statistical differences between the groups: any letter other than “a” signifies a difference from the control group; any letter other than “b” signifies a difference from the APAP group; and “c” signifies a significant difference from both the control group (“a”) and the APAP group (“b”), as determined by a one-way analysis of variance followed by Tukey’s post hoc test, with the significance set at *p* < 0.05. The statistical contingency analysis in panels (**F**) and (**G**) was assessed using the chi-square test and Fisher’s exact test, with the different letters indicating statistical differences between the groups. Black arrows indicate area of necrosis.

Figure 2E shows the quantitative analysis of the necrotic area, along with a semi-quantitative analysis of the hyperemia (Figure 2F) and binucleation (Figure 2G) indices. An increase in the necrotic area is observed in the APAP group compared to the control group. Both the P20 and P40 groups exhibit smaller necrotic areas than the APAP group, and all the groups show significant differences compared to the control group.

For the binucleation index, piperine at 20 mg/kg preserved the hepatocyte regeneration index, which was statistically equivalent to that of the control group. The APAP group showed a decrease in the binucleation index, which differed from that of the control and P20 groups. Piperine at 40 mg/kg showed no significant difference compared with the other groups (Figure 2F).

Both the APAP and P20 groups demonstrated an increase in the hyperemia index, which was significantly different from that of the other groups. No significant differences were observed between the P40 and control groups (Figure 2G).

Figure 2A–D provide photomicrographs that offer a qualitative representation of the necrotic areas. Intact hepatocytes and vesicular nuclei are observed in the control group (Figure 2A). Figure 2B shows increased necrosis in the centrilobular region of the animals intoxicated with APAP, and in the P20 and P40 groups (Figure 2C,D), the hepatocytes display greater integrity in their cellular structure.

### 2.3. Piperine Prophylaxis Reduces Hepatic Oxidative Damage

**Figure 3:**
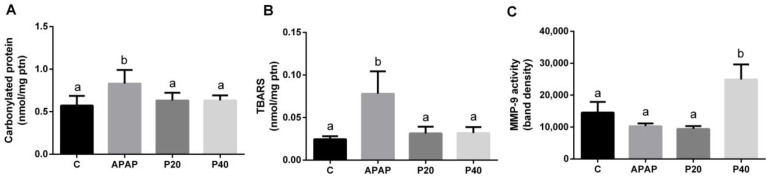
Assessment of oxidative damage biomarkers. Liver damage was evaluated by measuring protein carbonylation (**A**) and thiobarbituric acid reactive substances (TBARS) (**B**), while matrix remodeling was assessed via matrix metalloproteinase 9 (MMP-9) activity in zymography assays (**C**) of the livers of C57BL/6 mice subjected to prophylactic piperine treatment for 8 days, followed by APAP intoxication. The different letters indicate statistical differences between the groups. In panels A and B, any letter other than “a” signifies a difference from the control group, and any letter other than “b” signifies a difference from the APAP group. In panel C, the letter “b” indicates that the P40 group is different from the other experimental groups, as determined by a one-way analysis of variance followed by Tukey’s post hoc test. Statistical significance is considered at *p* < 0.05.

For the biomarker profile associated with protein and lipid oxidation, we observed increased levels of carbonylated proteins (Figure 3A) and thiobarbituric acid reactive species (TBARS) (Figure 3B). Prophylaxis with both doses of piperine was effective for maintaining carbonylated protein and TBARS levels, which were statistically equivalent to those in the control group. We also assessed the activity of the proteolytic enzyme metalloproteinase 9 (MMP-9) (Figure 3C), and observed a significant increase in the P40 group compared to that in the other groups.

### 2.4. Piperine Prophylaxis Modulates Expression of Cytochrome P450 Isoenzymes and Inflammasome Pathway 

**Figure 4:**
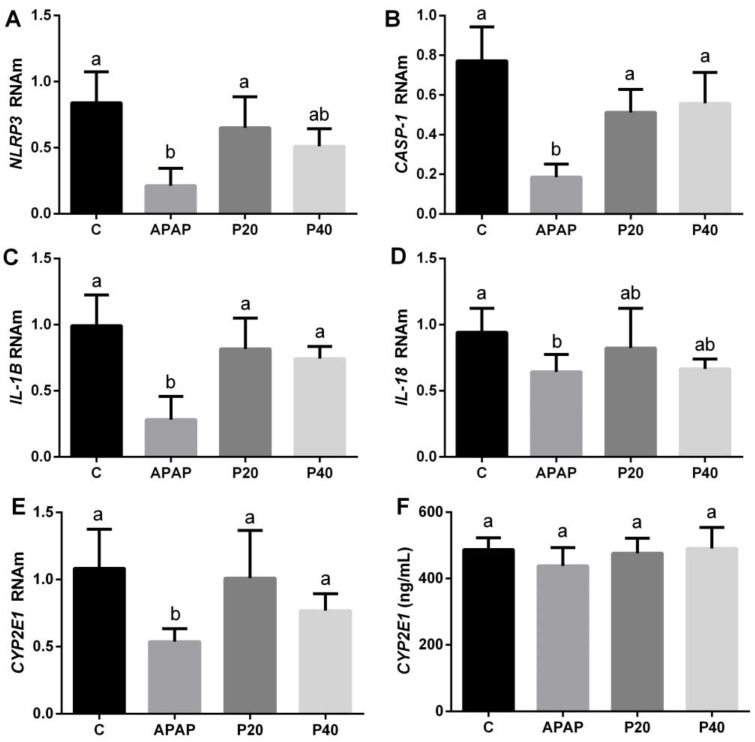
Evaluation of gene expression in the inflammasome pathway (*NLRP3* (**A**)*, CASP-1* (**B**)*, IL-1β* (**C**), and *IL-18* (**D**)) and the isoenzymes *CYP2E1* (**E**) and *CYP1A2* (**F**) in the mice subjected to prophylactic treatment with piperine for 8 days, followed by APAP intoxication. The different letters indicate statistical differences between the groups: any letter other than “a” signifies a difference from the control group, and any letter other than “b” signifies a difference from the APAP group. The notation “ab” is used when the statistical analysis indicates that a group is statistically equal to both the control and APAP groups, as determined by a one-way analysis of variance followed by Tukey’s post hoc test. Abbreviations: *NLRP3*: NLR family pyrin domain-containing protein 3; *CASP-1*: caspase-1; *IL-1β*: interleukin-1β; *IL-18*: interleukin-18; *CYP1A2*: cytochrome P450 1A2; *CYP2E1*: cytochrome P450 2E1.

Inflammasomes are responsible for activating the inflammatory process. Therefore, we evaluated the profiles of the genes expressed in the inflammasome pathway. We observed a difference in the APAP group, with a decrease in the *NLRP3* gene compared to that in the control and P20 groups (Figure 4A). The gene expression of *caspase-1* (Casp-1) and *interleukin-1β* (IL-1β) decreased only in the APAP group, and was statistically different from the control and prevention groups (P20 and P40), which maintained levels similar to those of the control (Figure 4B,C). *Interleukin 18* (IL-18) decreased in the APAP group, showing a statistically significant difference compared with all the other groups (Figure 4D).

During APAP metabolism, cytochrome P450 plays an important role in the formation of the toxic adduct NAPQI, and the CYP2E1 and CYP1A2 enzymes are responsible for increasing systemic exposure to the drug. Thus, we observed a decrease in the mRNA level of *CYP2E1* in the APAP group, showing a significant difference when compared to the control, P20, and P40 groups (Figure 4F). As for *CYP1A2*, no statistically significant difference was observed between the groups and the control (Figure 4E).

### 2.5. Serum and Liver Inflammatory Profiles 

**Figure 5:**
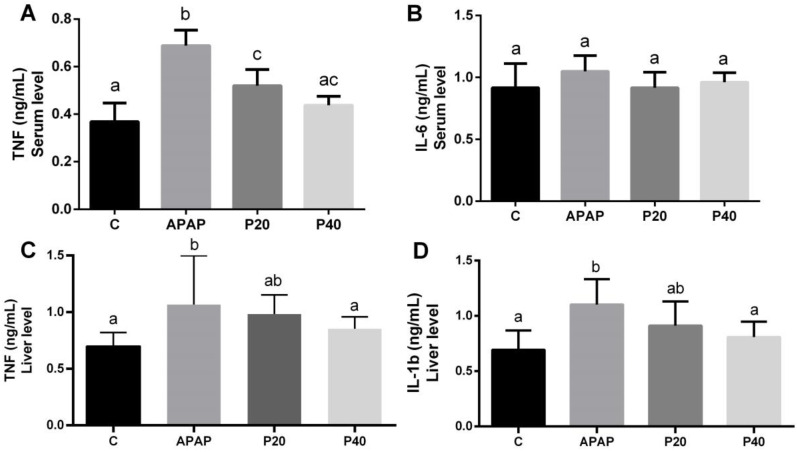
Evaluation of the cytokines TNF-α (**A**) and IL-6 (**B**) in the serum, and TNF-α (**C**) and IL-1β (**D**) in the liver of mice subjected to prophylactic treatment with piperine for 8 days, followed by APAP intoxication. The different letters indicate statistical differences between the groups: any letter other than “a” signifies a difference from the control group, any letter other than “b” signifies a difference from the APAP group, and “c” indicates that the group is significantly different from both the control group (“a”) and the APAP group (“b”). The notation “ac” is used when the statistical analysis shows that the group is statistically similar to both the control and P20 groups. The notation “ab” is used when the statistical analysis indicates that the group is statistically equal to both the control and APAP groups, as determined by a one-way analysis of variance followed by Tukey’s post hoc test. Significant differences are considered at *p* < 0.05. Abbreviations: TNF-α: tumor necrosis factor-alpha; IL-6: interleukin-6; IL-1β: interleukin-1 beta.

To evaluate the systemic inflammatory profile, the levels of the cytokines tumor necrosis factor-alpha (TNF-α) and interleukin-6 (IL-6) in the serum of the animals were assessed. In the APAP group, the TNF-α levels were significantly increased compared to the other experimental groups (Figure 5A). The P20 group exhibited a significant difference from both the control and APAP groups, while the P40 group showed no differences from either the control or the P20 groups. 

No statistically significant differences were observed among the experimental groups for IL-6 (Figure 5B). 

To evaluate the liver inflammatory profile, the levels of the cytokines tumor necrosis factor-alpha (TNF-α) and interleukin-1 beta (IL-1β) were assessed in the livers of the animals. In the APAP group, the TNF-α levels were significantly increased compared to the control and P40 groups (Figure 5C). The P20 group, however, displayed values statistically equivalent to both the control and APAP groups. In the P40 group, we observed a significant reduction compared to the APAP group, which was statistically equivalent to the control group. 

A similar pattern was observed for the IL-1β levels in the livers (Figure 5D).

## 3. Discussion

Piperine can plays an important role because of its antioxidant, hepatoprotective [24], anti-inflammatory [25], and other benefits. Our study aimed to evaluate the hepatoprotective capacity of piperine administered before paracetamol intoxication and investigate its hepatoprotective effect against drug-induced injuries. Paracetamol administered at a dose of 500 mg/kg exhibits high toxicity in mice [27,28,29]. Therefore, we investigated the prophylactic potential of piperine for mitigating liver damage induced by drug intoxication. The findings revealed the effectiveness of piperine at both doses for preventing liver damage, offering protection against necrosis and oxidative stress. We observed an increase in ALT levels within the initial 3 h after APAP administration. Notably, the prevention groups administered piperine at 20 mg/kg (P20) and 40 mg/kg (P40) maintained ALT levels comparable to those in the control group. In addition, a significant increase was observed in the hepatocyte necrosis parameters in the group exposed only to paracetamol (APAP), whereas the groups treated with piperine at both doses were protected from necrosis. This suggests that piperine protects the liver by preventing cell death caused by necrosis, an event characteristic of paracetamol intoxication [30]. These results are in line with the data in the literature describing the hepatoprotective properties of piperine [24,31].

Hepatocyte regeneration is crucial for the renewal of liver cells, particularly in situations that induce liver damage [32]. The binucleation of hepatocytes indicates cell proliferation. We observed that the groups pretreated with piperine at doses of 20 mg/kg (P20) and 40 mg/kg (P40) exhibited results similar to those of the control group. Conversely, during the initial 3 h period after paracetamol intoxication (APAP), we noted a reduction in cell division, which is consistent with previous findings described in the literature [29]. Paracetamol-induced drug intoxication contributes to a redox imbalance through the formation of the toxic adduct NAPQI when APAP is metabolized by cytochrome P450 (CYP450) [33,34,35]. In our study, we observed a significant increase in carbonylated protein and TBARS in the APAP group, whereas the levels in the groups pretreated with piperine (P20 and P40) were equal to those in the control group. These data suggest that piperine protects against oxidative damage to proteins and lipids during paracetamol-induced drug intoxication [26,36]. When controlled, inflammation is a beneficial response of the body to tissue damage [15]. Metalloproteinases are gelatinases that are activated during inflammation and regulate various biological processes [37]. The literature shows that the proteolytic activity of metalloproteinase 9 (MMP-9) increases during the acute phases of inflammation, suggesting its role as a biological barrier in the early stages and its potential protective function in certain pathological situations, such as colitis-associated cancer [38]. Therefore, MMP-9 may play a paradoxical role in the liver, with both beneficial and harmful effects. For instance, in cases of ischemia or perforation injuries, MMP-9 has been observed to favor liver recovery [39]. In our study, an increase in MMP-9 was observed only in the group pretreated with 40 mg/kg piperine, suggesting that prophylaxis with piperine at the highest dose may induce a beneficial adaptive response to APAP insult. In addition, hyperemia was reduced only in the P40 group. Thus, we propose the control of tissue blood vascularization. Increased blood vascularization is directly linked to increased inflammation and the red blood cell count [32]. 

Next, we investigated the expression of the inflammasome pathways. During paracetamol-induced hepatotoxicity, inflammasome activation contributes to the worsening of liver injury [40]. Inflammasome activation is described as a two-signal process. The first, known as the ‘priming signal’, initiates the transcription of the precursors for pro-inflammatory proteins, such as pro-IL-1β and NLRP3 itself, although it does not necessarily lead to their immediate translation. A second step, referred to as the ‘activation signal’, is required for the cleavage of pro-IL-1β into its active form, IL-1β, promoting the release of this cytokine [41]. This activation signal involves damage-associated molecular patterns (DAMPs) generated by endogenous stress, which trigger inflammatory pathways aimed at tissue repair [42].

The expression of the *NLRP3*, *CASP-1*, *IL-1β*, and *IL-18* genes in the piperine-preventive groups (P20 and P40) remained at the same levels as the control, suggesting the immunomodulatory potential of piperine. Furthermore, we can infer that 3 h after intoxication with paracetamol was not sufficient to detect an increase in gene expression in the inflammasome pathway, as previous studies have suggested that an increased expression of these components is observed at longer times, such as 12 or 24 h after intoxication [29,43,44]. 

During acetaminophen toxicity, oxidative damage is suggested to increase, as evidenced by increased carbonylated proteins (Figure 3A) and TBARS (Figure 3B). This oxidative stress, along with a greater necrotic area (Figure 2E), serves as a stimulus for the second phase of inflammasome activation, activating caspase-1 and leading to the cleavage and release of IL-1β, independent of changes in RNA expression. Piperine pretreatment appears to modulate this second phase of inflammasome activation. Although piperine does not alter *IL-1β* or *NLRP3* mRNA levels, it appears likely that it reduces oxidative stress (Figure 3) and cellular necrosis (Figure 2), limiting caspase-1 activation and, consequently, the release of IL-1β. Thus, we infer that RNA levels remain stable relative to those of the control, while the conversion of pro-IL-1β to active IL-1β is reduced due to piperine’s antioxidant and anti-inflammatory effects. These findings are supported by the literature, which suggests that, in response to stress, there may be a discrepancy between mRNA levels and the active forms of cytokines at the site of inflammation. For instance, in liver injury models, tissue damage often correlates with increased inflammatory protein levels, while RNA levels may decrease as part of compensatory mechanisms for tissue regeneration [45].

Piperine and paracetamol are metabolized via the cytochrome P450 pathway [46]. Thus, we evaluated the gene expression of the *CYP2E1* isoform and observed a reduction in gene expression in the APAP group, suggesting an adaptive response of the cells to control the toxicity induced by the formation of the toxic adduct NAPQI [47,48]. In contrast, in the P20 and P40 groups, in which piperine was administered preventatively, the expression of *CYP2E1* was statistically similar to that in the control group. This aligns with the observed profile for the measurement of carbonylated proteins, TBARS, and the expression of the inflammasome pathway.

**Figure 6:**
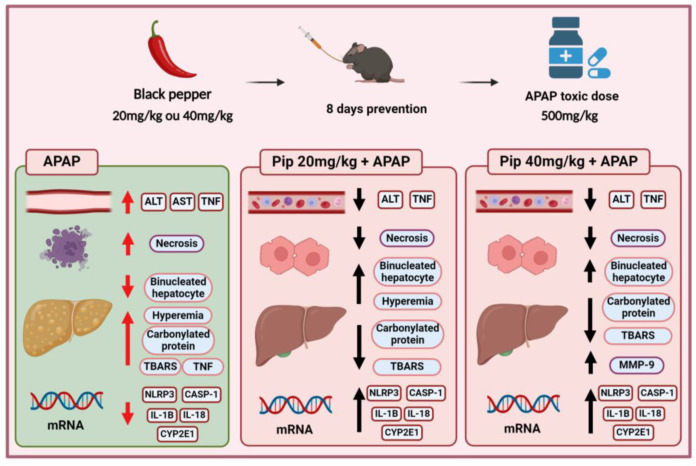
Piperine administered preventively for 8 days protected against drug intoxication induced by paracetamol. Parameters, including biochemical, histological, redox status, and inflammatory, were preserved in piperine-treated groups, indicating liver protection. The red arrows represent the effects of paracetamol on the different parameters evaluated, while the black arrows represent the effects of the P20 and P40 groups on these parameters.

Tumor necrosis factor-alpha (TNF-α) is one of the main cytokines involved in cell death signaling in the liver [16,49,50]. In the group exposed to paracetamol alone (APAP), a significant increase was observed in the level of TNF-α, indicative of liver intoxication and hepatocyte necrosis induction. This profile was similarly observed systemically. However, in the groups pretreated with piperine at both doses, the increase in TNF-α was lower compared to the APAP group, suggesting a potential anti-inflammatory effect of piperine as part of its preventive mechanism. These results suggest that piperine, administered beforehand, can attenuate the TNF-mediated inflammatory response during liver intoxication, which may contribute to protecting the liver against cell damage and necrosis. Thus, piperine may play a role in the prevention of hepatic alterations and a hepatoprotective role in drug intoxication (Figure 6).

## 4. Materials and Methods

### 4.1. Reagents

The chemicals that were used include distilled water, free water, piperine (Sigma-Aldrich, St. Louis, MO, USA), paracetamol (EMS), IGEPAL^®^ CA-630 (Sigma-Aldrich, St. Louis, MO, USA), sodium deoxycholate, carboxymethylcellulose (Sigma-Aldrich, St. Louis, MO, USA), buffered formalin, alcohols, paraffin, hematoxylin, eosin, potassium phosphate, EDTA, SDS, protease inhibitor (PMSF) (Sigma-Aldrich, St. Louis, MO, USA), Triton X-100 (Sigma-Aldrich, St. Louis, MO, USA), Coomassie Brilliant blue G-250 (Sigma-Aldrich, St. Louis, MO, USA), methanol (Neon), acetic acid, Tris, NaCl, CaCl_2,_ and NaN_3_. Analytical-grade chemicals were used in this study.

### 4.2. Animals

This study was approved by the Ethics Committee for the Use of Animals (CEUA) of the Federal University of Ouro Preto (UFOP) under protocol number 5402121118. Isogenic C57BL6 male mice were housed in cages at the UFOP Animal Science Center (CCA) under controlled humidity and temperature. The animals were between 9 and 11 weeks old, weighed an average of 23–25 g, and were subjected to 12 h light/dark cycles with ad libitum access to water and food.

### 4.3. Experimental Design

The animals received piperine orally once a day at doses of 20 mg/kg or 40 mg/kg diluted in carboxymethylcellulose (0.5%) for 8 consecutive days without fasting, according to previous laboratory studies [51]. On the 8th day of piperine administration, after a 12 h interval, the animals were subjected to APAP intoxication (dose of 500 mg/kg). The animals were anesthetized by the inhalation of isoflurane (Isoforine^®^). They were euthanized 3 h after APAP administration by exsanguination via cardiac puncture. The control group (C) received the vehicle carboxymethylcellulose (0.5%) for 8 consecutive days. Distilled water was added after 12 h. The APAP group received the vehicle carboxymethylcellulose (0.5%) for 8 consecutive days. After 12 h, a single dose of 500 mg/kg APAP was administered. The P20 + APAP groups received piperine (20 mg/kg) diluted in carboxymethylcellulose (0.5%) for 8 consecutive days. After 12 h, a single dose of 500 mg/kg APAP was administered. The P40 + APAP groups received piperine (40 mg/kg) diluted in carboxymethylcellulose (0.5%) for eight consecutive days. After 12 h, a single dose of 500 mg/kg APAP was administered.

### 4.4. Paracetamol-Induced Drug Liver Injury Model

Hepatotoxicity was induced in the C57BL/6 mice according to the APAP intoxication model using a non-lethal dose of 500 mg/kg and standardized in previous laboratory studies [27,28,29]. Paracetamol was obtained in liquid form from EMS Pharmaceutical Industry (batch OX0255, Hortolandia, SP, Brazil). To ensure gastric emptying, the animals fasted for 6 h and after this time, the toxic dose of APAP (500 mg/kg) was administered orally via an orogastric gavage in a volume of 120 µL. The animals in the control group received only 120 µL of distilled water orally.

### 4.5. Histological and Morphometric Analyses

To preserve the liver tissue, 4% buffered formalin was used for storage. The liver tissue was fixed, processed in an increasing series of alcohol (70–100%), followed by paraffin embedding. Paraffin sections of around 4 μm were obtained using a semi-automatic microtome and the slides were mounted and stained using hematoxylin and eosin (HE) staining. A Leica optical microscope (Leica DFC 300 FX; Leica Microsystems, Wetziar, Germany) coupled with a DM5000 digital camera and Leica Application Suite analysis software (Lecva application suite version 3) from the Advanced Microscopy and Microanalysis Multiuser Laboratory at the Biological Sciences Research Center of the Federal University of Ouro Preto were used to obtain the photomicrographs. Twenty images were obtained for each slide, corresponding to one animal, at 40× magnification. A quantitative morphometric analysis of the necrotic areas was performed using ImageJ software version 1.32j (National Institutes of Health, Bethesda, MD, USA). A qualitative analysis was performed using manual tools involving the number of pixels [17,29].

### 4.6. Biomarkers of Hepatic and Renal Injury

The serological samples were utilized to assess the serum levels of the liver enzymes ALT and AST. The commercial kits used were from LABTEST^®^ laboratory (LABTEST Diaginostica SA, Lagoa Santa, MG, Brazil), according to the protocols provided by the manufacturer.

### 4.7. Profile of Inflammatory Mediators

The ELISA immunoenzymatic method was used to analyze the cytokines using commercial kits from Peprotech^®^ (Rocky Hill, NJ, USA) and Biorbyte (Cambridge, UK). Liver and serum samples were utilized to measure the inflammatory cytokines TNF-α, IL-6, and IL-1β.

The kits used in this work were Murine TNF-α (catalogue #900-K54), Murine IL-6 (catalogue #900-K50), and Murine IL1-β (catalogue #900-k47), following the manufacturer’s instructions. Absorption readings were taken at 405 nm, with the wavelength correction set to 630 nm [52]. 

### 4.8. Biomarkers of Hepatic Oxidative Damage

The homogenate for the total protein, carbonylated protein, and thiobarbituric acid (TBARS) measurements was prepared using 100 mg of liver, 1 mL of 50 mM potassium phosphate buffer + 0.5 mM EDTA (pH 7.2), and 1 mM protease inhibitor (PMSF). The mixture was then centrifuged at 9300× *g* for 10 min at 4 °C to collect the supernatant. The Lowry method was used to determine the protein content [53]. 

The carbonyl protein dosage was used to quantify the oxidative damage to proteins, resulting in the formation of carbonyl groups. The carbonylated proteins were quantified according to the method described by Levine et al. [54] and adapted from previous laboratory protocols [55,56]. At this dose, the carbonyl proteins reacted with 2,4-dinitrophenylhydrazine (DNPH) to form hydrazones, which were measured using a spectrophotometer at an absorbance of 370 nm. The data were expressed as nmol/mg of protein. 

The detection of TBARS to determine the lipid peroxidation, serving as an indicator of oxidative damage to the lipids, was based on the method described by Draper et al. [57] and adapted from previous laboratory protocols [55,56]. For this dosage, 125 µL of trichloroacetic acid (TCA), 125 µL of thiobarbituric acid (TBA), and 62.5 µL of butylated hydroxytoluene (BHT) were added. The absorbance was measured using a spectrophotometer (Biospectro/SOS laboratory, MG, Brazil) at an absorbance of 532 nm.

### 4.9. Quantitative Real-Time PCR (qRT-PCR)

The primers used to amplify the selected genes were designed based on the mRNA sequences available in the Mouse Genome Database and with the help of NCBI/Primer-BLAST. The total RNA was extracted from the livers of the mice using an SV Total RNA Isolation System kit (Promega, Madison, WI, USA), according to the manufacturer’s instructions. The amount of extracted RNA was checked using a 260/280 wavelength ratio as an indication of purity, and its integrity was assessed on an agarose gel [58]. Complementary deoxyribonucleic acid (cDNA) was synthesized from 2 µg of the total RNA extracted using a Capacity cDNA Reverse Transcription Kit (Thermo Fisher, Waltham, MA, USA). The quantitative reverse transcription polymerase chain reaction (qRT-PCR) technique was used to analyze the gene expression of the genes under study. In 96-well plates, to obtain a final volume of 10 μL, 1 μL of cDNA diluted 5× in free water, 0.5 μL of each primer (forward and reverse, 10 μM), 5 μL Power of SYBR^®^ Green PCR Master Mix (Applied Biosystems, Foster, CA, USA), and 3 μL of DNAse-free water were inserted into each well. The qRT-PCR reaction was carried out using the programming contained in an Applied Biosystems ABI 7300 device (city, country), and the signals obtained were normalized using the levels of *β-actin* (*Actb*), the reference gene used. The primer sequences used were as follows: *CYP2E1*—F’TTTCCCTAAGTATCCTCCGTGAC, R’TCGTAATCGAAGCGTTTGTTG; *CYP1A2*—F’ACAAGACCCAGAGCGAGAAG, R’GCAGCAGGATGGCTAAGAAG; *NLRP3*—F’GGCGAGACCTCTGGGAAAAA, R’CCAGCAAACCCATCCACTCT; *Casp-1*—F’CTGGGACCCTCAAGTTTTGCC, R’GGCAAGACGTGTACGAGTGGT; *IL-1β*—F’AGAGCCCATCCTCTGTGACT, R’GGAGCCTGTAGGTGCAGTTGT; *IL-18*—F’ATTTTACTATCCTTCACCGAGAGG, R’TGTTCGAGGATATGACTGATATTGA; and *β-actin*—F’CACTGTCGAGTCGCGTCCA, R’TCATCCATGGCGAACTGGTG. From the SYBR^®^ Green fluorescence emission intensity during the exponential phase, the quantification cycle (Cq) was determined, and the Cq results were normalized using *β-actin*. Gene expression was calculated using the 2^−ΔΔCq^ method [59].

### 4.10. Zymography

The activity of metalloproteinase 9 (MMP-9) was determined by zymography, as previously described [25,26]. The tissue was homogenized and centrifuged at 10,000 g for 10 min at 4 °C, using RIPA buffer (pH 8.0) with 150 mM NaCl, 1% IGEPAL^®^ CA-630 (Sigma-Aldrich, Co, St Louis, MO, USA), 0.5% sodium deoxycholate, 0.1% SDS, and 50 mM Tris. The supernatant was collected for insertion into 8% polyacrylamide gels copolymerized with 2 mg/mL gelatin. After the run, the gels were washed in 2.5% Triton X-100 (3 × 20 min), and incubated for 18 h at 37 °C in a buffer comprising 50 mM Tris, 150 mM NaCl, 5 mM CaCl_2_, and 0.05% NaN_3_ (pH 7.5). The gels were stained with 0.05% Coomassie Brilliant Blue G-250 for 3 h and decolorized using an acid–alcohol solution (4% methanol–8% acetic acid). The bands were quantified using ImageJ software version 1.32j (National Institutes of Health, Bethesda, MD, USA), and the optical density was measured.

### 4.11. Statistical Analyses

G*Power software was used to determine the sample size. The sample size was calculated using a power of 0.90 and an alpha value of 0.05. The data were presented as the mean ± standard error. The statistical significance was set at *p* < 0.05. The Kolmogorov–Smirnov test was used to assess normality. The normally distributed data were analyzed using a univariate one-way ANOVA followed by Tukey’s post hoc test. For the data that did not show a normal distribution, a contingency analysis was performed with the data evaluated using Fisher’s exact test, and the results were expressed as a dot plot. The statistical analyses were performed using GraphPad Prism software (version 8.0, GraphPad Software Inc., San Diego, CA, USA).

## 5. Conclusions

The administration of piperine for eight consecutive days reduced paracetamol-induced liver injury by minimizing the extent of necrotic areas, decreasing ALT levels, reducing damage to proteins and lipids, modulating the genes involved in the inflammasome and cytochrome P450 pathways, and avoiding an increase in TNF and IL-beta in the liver, particularly at the highest dose. 

Thus, we conclude that the prophylactic administration of piperine as an herbal medicine can protect the liver against drug intoxication.

## Data Availability

The original contributions presented in this study are included in the article; further inquiries can be directed to the corresponding author/s.
